# MethylC-analyzer: a comprehensive downstream pipeline for the analysis of genome-wide DNA methylation

**DOI:** 10.1186/s40529-022-00366-5

**Published:** 2023-01-06

**Authors:** Rita Jui-Hsien Lu, Pei-Yu Lin, Ming-Ren Yen, Bing-Heng Wu, Pao-Yang Chen

**Affiliations:** 1 Institute of Plant and Microbial Biology, Academia Sinica, Taipei, 115 Taiwan; 2 Department of Medicine, Washington University in St. Louis, St. Louis, MO USA

**Keywords:** DNA methylation, Differentially methylated regions, MethylC-analyzer, Next-generation sequencing, Bisulfite sequencing, Whole-genome bisulfite sequencing, Reduced representation bisulfite sequencing, Enzymatic methyl-seq

## Abstract

DNA methylation is a crucial epigenetic modification involved in multiple biological processes and diseases. Current approaches for measuring genome-wide DNA methylation via bisulfite sequencing (BS-seq) include whole-genome bisulfite sequencing (WGBS), reduced representation bisulfite sequencing (RRBS), and enzymatic methyl-seq (EM-seq). The computational analysis tools available for BS-seq data include customized aligners for mapping bisulfite-converted reads and computational pipelines for downstream data analysis. Current post-alignment methylation tools are specialized for the interpretation of CG methylation, which is known to dominate mammalian genomes, however, non-CG methylation (CHG and CHH, where H refers to A, C, or T) is commonly observed in plants and fungi and is closely associated with gene regulation, transposon silencing, and plant development. Thus, we have developed a MethylC-analyzer to analyze and visualize post-alignment WGBS, RRBS, and EM-seq data focusing on CG. The tool is able to also analyze non-CG sites to enhance deciphering genomes of plants and fungi. By processing aligned data and gene location files, MethylC-analyzer generates a genome-wide view of methylation levels and methylation in user-specified genomic regions. The meta-plot, for example, allows the investigation of DNA methylation within specific genomic elements. Moreover, our tool identifies differentially methylated regions (DMRs) and investigates the enrichment of genomic features associated with variable methylation. MethylC-analyzer functionality is not limited to specific genomes, and we demonstrated its performance on both plant and human BS-seq data. MethylC-analyzer is a Python- and R-based program designed to perform comprehensive downstream analyses of methylation data, providing an intuitive analysis platform for scientists unfamiliar with DNA methylation analysis. It is available as either a standalone version for command-line uses or a graphical user interface (GUI) and is publicly accessible at https://github.com/RitataLU/MethylC-analyzer.

**Supplementary Information:**

The online version contains supplementary material available at 10.1186/s40529-022-00366-5.

## Background

DNA methylation, referring to the addition of a methyl group to the fifth carbon of cytosine (C) to form 5-methylcytosine (5mC), is one of the most crucial epigenomic mechanisms in biological processes. DNA methylation occurs in the symmetric CG and CHG contexts and in the asymmetric CHH context (where “H” represents A, C, or T) (Hsu et al. 2018). The biological processes known to be associated with DNA methylation include genomic imprinting, gene silencing, embryonic development, X chromosome inactivation, the alteration of chromatin structure, and transposon inactivation (Jones 2012; Wilson et al. 2012).

Experimental approaches such as methylated DNA immunoprecipitation sequencing (MeDIP-seq) (Weber et al. 2005), reduced representation bisulfite sequencing (RRBS) (Meissner et al. 2005), whole-genome bisulfite sequencing (WGBS) (Cokus et al. 2008), Illumina’s Infinium Methylation 450 K/EPIC BeadChip (Pidsley et al. 2016) and enzymatic methyl-seq (EM-seq) (Vaisvila et al. 2021) have been developed to measure genome-wide methylation. MeDIP-seq is an affinity enrichment-based approach that uses 5mC-specific antibodies to enrich methylated DNA fragments. Illumina’s Infinium HumanMethylation450 BeadChip (HM450K) relies on hybridization of genomic fragments to probes on the chip to measure the DNA methylation of 485,512 CpG sites in the human genome (Naeem et al. 2014). The Infinium Methylation EPIC BeadChip (EPIC) improved HM450 array covers over 850 K CpG sites, including > 90% of the CpGs from HM450 and an additional 413,743 CpGs. RRBS and WGBS both rely on the bisulfite conversion-based method. During bisulfite conversion, unmethylated cytosines (C) are converted to uracils (U), while the methylated cytosines do not react with sodium bisulfite and thus remain unchanged. As the converted uracils (U) are turned into thymines (Ts) during PCR amplification, all the Cs in the corresponding sequencing reads represent 5mCs in the sample DNA (Frommer et al. 1992). WGBS theoretically covers all cytosines in the genome, and RRBS targets enriched CpG-rich regions (Gu et al. 2011). Likewise, in addition to sodium bisulfite, DNA bases can also react with enzymes such as ten-eleven translocation (TET) family enzymes and APOBEC2. The EM-seq technique detects 5mC and 5hmC using two sets of enzymatic reactions. In the first enzymatic step, TET2 is applied to oxidize methylated cytosines, and APOBEC2 is used to deaminate unmethylated cytosine (C) to uracil (U). After PCR amplification, oxidized methyl cytosines form base pairs with guanines (G), and uracils (U) pair with adenines (A). BS-seq approaches, including WGBS and RRBS, are commonly used for quantifying DNA methylation with single-base resolution. Since the end products of WGBS and EM-seq are the same, the same analysis tools can be used.

Several computational tools have been created for BS-seq analysis. For example, BAT is a toolkit that facilitates bisulfite sequencing data analysis, providing standard processing and analysis steps from raw read alignment up to the calculation of DMR correlations (Kretzmer et al. 2017). Bicycle is a command-line-based tool that uses raw read alignments to identify differentially methylated sites in WGBS, RRBS, and hydroxymethylation datasets (Grana et al. 2018). RnBeads 2.0 is a R/Bioconductor package and can perform differential DNA methylation analysis on EPIC microarray and BS-seq data. RnBeads 2.0 improves upon the original version with enhanced computational efficiency and the addition of an intuitive graphical user interface (GUI) (Muller et al. 2019). nf-core/methylseq is a workflow management system built by Nextflow that can run tasks across multiple computational infrastructures in a portable manner. It focuses on processing, from raw reads to methylation site calling (Ewels et al. 2020). Although many tools are available to handle BS-seq data, there is still a gap in pipeline automation with customizable downstream analyses for users’ own datasets. Some tools require users to work with only the command-line interface (CLI) on Linux-like systems, which can be difficult for beginners, while others do not provide comprehensive downstream analysis or visualization functions. Here, we developed MethylC-analyzer, a comprehensive pipeline designed for the integrated analysis of BS-seq and EM-seq, enabling downstream analyses of post-alignment BS-seq and EM-seq for three kinds of cytosine methylation sites (CG, CHG, and CHH). MethylC-analyzer performs differential methylation analysis, including the identification of differentially methylated regions (DMRs) and differentially methylated genes (DMGs). Furthermore, it permits the investigation of locations that are enriched in these variable methylation regions. Finally, MethylC-analyzer comes with a GUI to provide an intuitive user experience.

### Construction and content

MethylC-analyzer is a Python-based pipeline incorporating R that performs various BS-seq and EM-seq analyses. The pipeline can be divided into three major components, (1) preprocessing, which generates intermediate outputs before analysis, such as summary tables of the methylation levels in each comparable region of aligned BS-seq datasets; (2) identifying differential methylation analysis, which compares methylation levels between two groups at the level of the whole genome or gene bodies; and (3) visualizations, where MethylC-analyzer will generate high quality figures (300 dpi) for each analysis step.

**Fig. 1:**
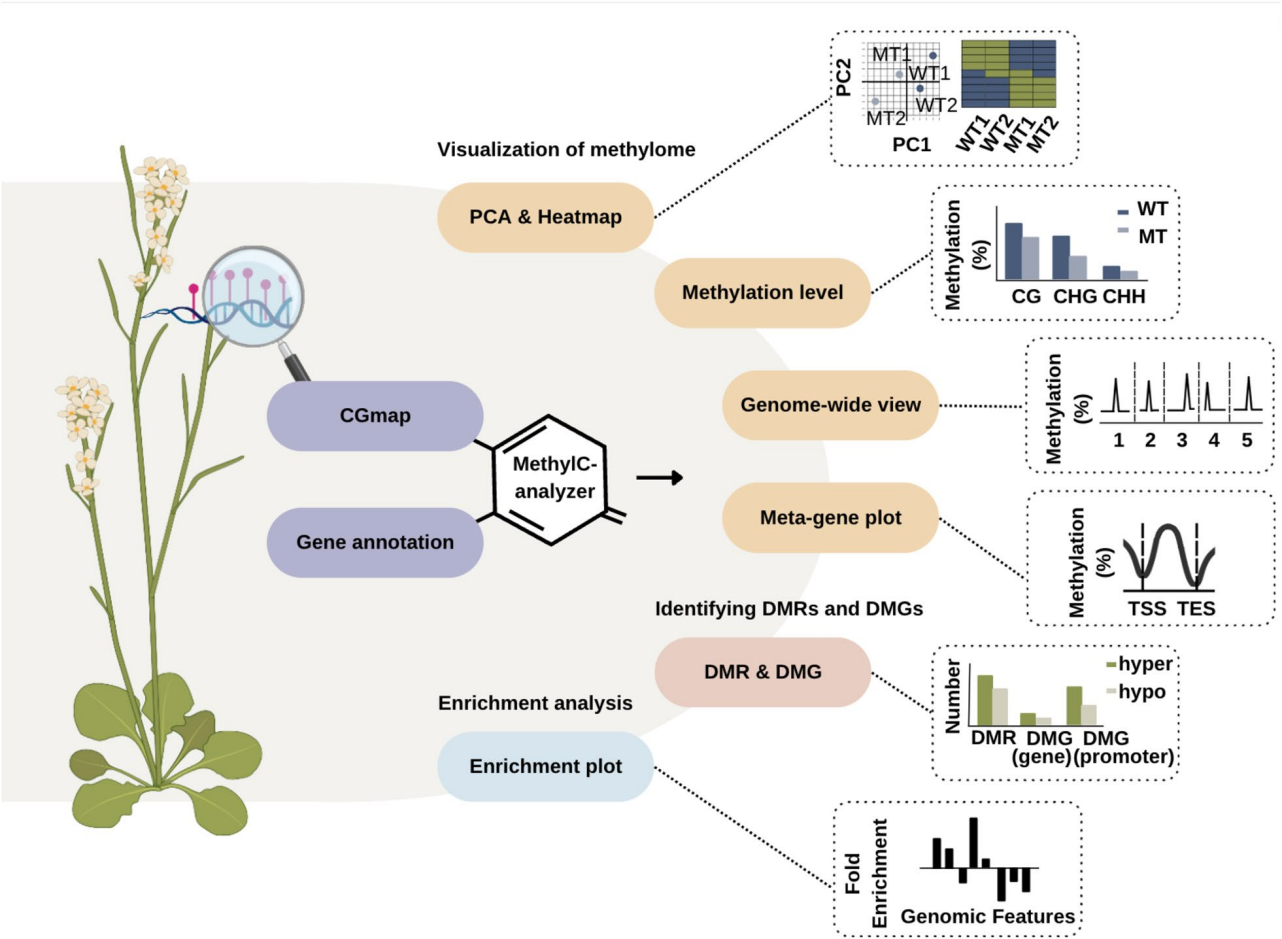
Schematic diagram of MethyC-analyzer. MethylC-analyzer is a sequential pipeline for analyzing post-align BS-seq. To run MethylC-analyzer, users provide a description text file with post-alignment methylation summaries for each cytosine site of samples, e.g. CGmap and gene annotation file (GTF). The first step MethylC-analyzer is to generate summary methylome figures of CG, CHG, and CHH context, including PCA, heatmap, and the distribution of methylation in each chromosome and gene-centric manner. The following is to perform differential methylation analysis between two groups, for example, identifying differential methylation regions (DMRs) and its related genes (DMGs). Also, comparing methylation status around specific regions, such as gene bodies and promoters. Moreover, to survey the DMR enrich status at specific genomic feature regions (i.e., gene bodies, promoters, exons, introns, S'UTR, 3'UTR, IGR.); The last step is to visualize the above analyses, MethylC-analyzer will generate publication-ready figures for each step

MethylC-analyzer is available as both a graphical user interface (GUI) and a standalone version for command-line usage. A list describing sample names and their corresponding post-alignment data (i.e., a CGmap) and gene annotation file (GTF) are essential inputs for MethylC-analyzer (Fig. 1). MethylC-analyzer can be executed in the local Unix/Linux environment, and a tutorial is provided at the GitHub repository (https://github.com/RitataLU/MethylC-analyzer). Alternatively, MethylC-analyser also incorporates the docker container, a lightweight virtualization technology, with its required system environment setting in terms of computing form. It offers a simplified and user-friendly way to start and deploy applications. The tutorial can be accessed via DockerHub https://hub.docker.com/r/peiyulin/methylc.

### Utility and discussion

#### Preprocessing of methylome data

The inputs of MethylC-analyzer are a list of samples and their corresponding post-aligned DNA methylation profiles (CGmap) and gene annotation file (GTF format). CG map (see Additional file 3: Table S1 for the format) provides sequence context for each cytosine (CG, CHG, CHH, H=A, T, or C) and estimates the DNA methylation level of cytosines in the reference genome, which is comparable to the output of bisulfite-specific aligners such as BS-Seeker2 (Guo et al. 2013), BS-Seeker3 (Huang et al. 2018) and BiSulfite Bolt (Farrell et al. 2021). The methylation calling files from other aligners can be converted to CG map format by MethylC-analyzer. MethylC-analyzer provides an independent Python script (see GitHub for methcalls2CGmap.py) to convert these files to compressed CGmap files. The output files from other aligners that can be handled by MethylC-analyzer include CX report files generated by Bismark, methylation calls generated by methratio.py in BSMAP (v2.73), and TSV files exported from the methylation calling status with METHimpute (Krueger and Andrews 2011; Li and Li 2009; Taudt et al. 2018). MethylC-analyzer utilizes the CG maps and adopts nonoverlapping window-based methods for genome-wide screening to compute the average methylation level. The eligible regions should contain at least four cytosines within 500 bp, with each cytosine covered by at least four reads in all samples by default; users have the flexibility to adjust these parameters.

#### Visualization of the general DNA methylome

MethylC-analyzer provides three kinds of figures for the visualization of overall DNA methylation patterns. First, MethylC-analyzer conducts principal component analysis (PCA) to condense the information of each sample into two dimensions for easy visualization. Each dot on the PCA plot indicates one sample, and the distance between dots represents the variation among samples. The closer any two dots are, the higher their similarity. Moreover, MethylC-analyzer generates a hierarchical clustering heatmap to represent methylation levels in a color scale. Each column indicates one sample, and each row represents one variable methylation region, where the difference in methylation between the maximum and minimum is 20%; the criterion for methylation differences indicative of a variable methylation region can be manually modified by the user. Furthermore, our tool evaluates the mean methylation levels within groups in three cytosine contexts (CG, CHG, CHH) and generates a bar plot representing the average methylation levels across groups in three contexts.

#### Visualization of DNA methylation across the whole genome and specific genomic regions

To visualize the genome-wide distribution of DNA methylation, MethylC-analyzer separates the genome into several large regions by using nonoverlapping tiling windows. By default, each window size is 1000 kb and covers at least four cytosines. Next, figures are generated to display the methylation levels of individual samples and the difference in methylation between the two groups across the genome at CG, CHG and CHH sites. Furthermore, MethylC-analyzer generates a metaplot encompassing regions 2 kb downstream and upstream of the gene body to investigate the DNA methylation levels proximal to the gene of interest.

#### Identifying differentially methylated regions and differentially methylated genes

To reveal the distinct methylation patterns between two groups, differentially methylated region analysis (DMR) is performed by comparing the average methylation levels between the control and experimental samples. Three different statistical testing methods are available for users to choose: Student’s t test, the Kolmogorov–Smirnov test (Massey 1951), and the Mann–Whitney U test (Mann and Whitney 1947). As a default setting, DMRs are defined as regions in which the difference in average methylation level is ≥ 10% with a p value less than 5%. Users have the flexibility to adjust these parameters. We provide both DMR text and BED files, and the BED files can be directly loaded into genome browsers such as the Integrative Genomics Viewer (IGV) as tracks for visualization (Robinson et al. 2017). A gene with a DMR located either in gene bodies or promoters will be considered a differentially methylated gene (DMG). The regions 2 kb upstream of transcriptional start sites (TSSs) are defined as promoters in our tool.

#### Enrichment analysis

To investigate whether DMRs are enriched or depleted in any specific genomic feature (e.g., promoter), MethylC-analyzer processes gene annotation GTF files into 8 genomic features as BED files of their locations, including promoter, gene body, exon, intron, 5′ untranslated region (5′UTR), coding sequence (CDS); 3′ untranslated region (3′UTR), and intergenic region (IGR). Then, the enrichment of DMRs at these genomic features against the genome background is calculated to produce a bar chart using the equation below:

$${log}_{2}\frac{\mathrm{RG}}{\mathrm{LR}} - {log}_{2}\frac{\mathrm{LF}}{\mathrm{LG}},$$

where RG is the length of the DMRs of a specific genomic feature, and LR is the total genomic length of all DMRs. This first ratio is used to estimate the percentage of specific genomic feature DMRs length among all DMRs in the genome. To evaluate the genome wide enrichment of the genomic feature, the percentage of the feature in the genome is accounted for, Hence, a second ratio between LF, the total length of this specific genomic feature, and LG, the length of the whole genome, is used for normalization. A higher enrichment value indicates that a DMR is more enriched in the specific genomic feature.

#### Demonstrating MethylC-analyzer on *Arabidopsis thaliana* WGBS

**Fig. 2:**
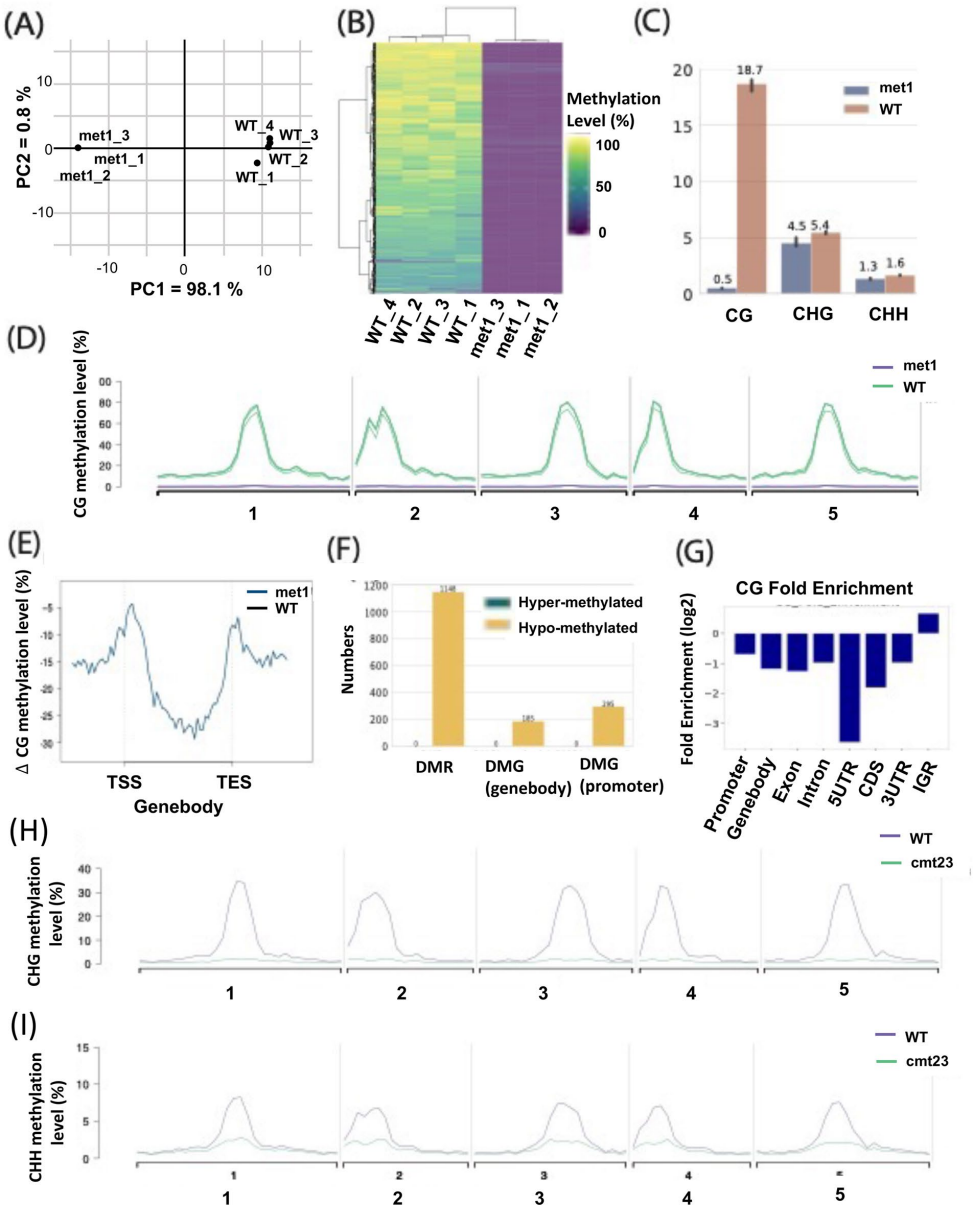
Visualization of Arabidopsis genomewide methylation analysis (**A**) PCA and (**B**) Hierarchical clustering showed a clear difference between met1 and wild-type (WT). **C** The average methylation level in two genotypes in 3 contexts (CG, CHG, CHH). **D** The CG methylation level in genome-wide view. **E** Metagene plot of Δ CG methylation levels in met1. **F** The summary of DMR and DMG numbers. **G** DMRs enriched in IGR. The CHG (**H**) and CHH (**I**) methylation level of cmt2 cmt3 double mutant and wild-type in genome-wide view. *TSS* transcription start site, *TES* transcription end site, *5′UTR* 5*′* untranslated region, *CDS* coding sequence, *3’UTR* 3′ untranslated region

To demonstrate that MethylC-analyzer on plants genomes, we downloaded and processed *Arabidopsis thaliana* (GSE122394, GSE148753) BS-seq datasets (Choi et al. 2020; Parent et al. 2021). The Arabidopsis WGBS included methyltransferase 1 (*MET1*) mutant, CMT2 CMT3 double mutant (*cmt2 cmt3*) and wild type (WT) strains. In the model plant Arabidopsis, CG methylation is maintained primarily by *MET1* (Law and Jacobsen 2010; Stroud et al. 2014); CMT2 and CMT3 play an important role in maintaining CHG and CHH methylation, respectively (Stroud et al. 2014; Zhong et al. 2021). We applied MethylC-analyzer to analyze four WT and three DNA methyltransferase met1 mutant strains (GSE122394) of *Arabidopsis thaliana*. These seven WGBS datasets were mapped to the *Arabidopsis* TAIR10 genome using BS-Seeker2 (Guo et al. 2013). The output files include a CG map and post-aligned mapping summaries for each cytosine, including coverage and methylation level. The reported DNA methylation level ranges from 0 (completely unmethylated) to 1 (fully methylated). A list of CG maps for each sample and a gene annotation file (GTF) were loaded into MethylC-analyzer for downstream analyses. First, MethylC-analyzer utilized CG maps and conducted nonoverlapping window-based methods for genome-wide screening and computed the average methylation level. The eligible regions contained at least four cytosines within 200 bp, with each cytosine covered by at least four reads in all samples. In total, there were 4895 regions qualified for CG methylation analysis. To visualize the general methylomes in *met1* and WT samples on CG methylation, MethylC-analyzer conducted principal component analysis (PCA) to generate a plot to show the variation between the two samples. PCA showed clearly separated the *met1* and WT samples. Based on principal component 1 (PC1), 98.1% of the windows were found to change their methylation state along with *met1* mutation (Fig. 2A), which is also supported by unsupervised hierarchical clustering analysis (Fig. 2B). The hierarchical clustering heatmap (Fig. 2B) showed 1102 variable CG regions in 7 samples; the DNA methylation level difference between maximum and minimum samples in each region was at least 50%. There is a clear difference in methylation between *met1* and WT, with *met1* exhibiting decreased DNA methylation relative to WT. Overall, the average CG methylation were 18.7% and 0.5% in WT and *met1*, respectively. *met1* samples were hypomethylated by 18.1% in comparison to the WT reference genome, and minor hypomethylation occurred in non-CG sites (Fig. 2C), indicating that *met1* corresponded with a nearly complete loss of CG methylation and a partial loss of non-CG methylation (Zhong et al. 2021).

To further investigate the distribution of the DNA methylation level and differences on each chromosome, we computed the methylation level along the whole genome in windows of 1 Mb and observed the distribution of *met1* and WT samples on the chromosomes. Figure 2D shows that the CG methylation could further contextualize the above results, as CG methylation was evenly decreased on all chromosomes in each *met1* sample (Fig. 2D). To investigate the DNA methylation distribution in the two groups in a gene-centric manner, MethylC-analyzer processed regions upstream of transcriptome start sites (TSSs), downstream of transcriptome end sites (TESs), and within gene bodies. In Fig. 2E, an increasing CG methylation level was observed at TSSs and TESs, whereas decreased methylation was observed within gene bodies in *met1* compared to WT. A 20% difference in DNA methylation (p value < 0.01) within 200 bp was considered a significant DMR (see section “Construction and content”). A total of 1148 hypo CG-DMRs and 0 hyper DMRs were identified (Fig. 2F), which matched the trend of decreased global methylation levels observed in the genome-wide methylation view (Fig. 2D). In total, 480 genes were associated with DMRs at a promoter or gene body (Fig. 2D) and were considered differentially methylated genes (DMGs). Then, we investigated whether these DMRs were localized to specific genomic regions or were randomly distributed. As shown in Fig. 2G, CG DMRs were enriched in the intergenic region (IGR) (Fig. 2G).

To decipher the non-CG methylation alteration in plant’s genome, we then applied MethylC-analyzer to *cmt2 cmt3* double mutant (GSE148753). One *cmt2 cmt3* double mutant of *Arabidopsis thaliana* was processed to evaluate the distribution of non-CG methylation in this line compared to that in one WT (GSE122394)*.* As shown in Fig. 2H, I, the *cmt2 cmt3* mutant depleted CHG and CHH methylation compared to the WT. This result is consistent with the previous findings that *cmt2* mutant of Arabidopsis reducing global CHH methylation; *cmt3* mutants lost CHG methylation, and *cmt2 cmt3* double mutants were stronger loss of CHG methylation than *cmt3* mutant (Stroud et al. 2014). The results from *met1* and *cmt2 cmt3* demonstrated that MethylC-analyzer is able to assess both CG and non-CG methylation.

#### Demonstrating MethylC-analyzer with human RRBS data

To demonstrate that MethylC-analyzer is applicable to a variety of genomes, we then downloaded and processed human (GSE110057) data (Bowden et al. 2020; Zhong et al. 2021). The human data were assessed using RRBS, and the library was generated from autosomal dominant polycystic kidney disease (ADPKD) and non-ADPKD kidney tissues, each of which had 3 replicates. ADPKD is the most common inherited kidney disease, affecting 1–5 per 10,000 individuals (Solazzo et al. 2018), and aberrant DNA methylation patterns are associated with many types of cancers. A previous study identified global hypermethylation in the genome of ADPKD-derived DNA (Woo et al. 2014), We performed all analyses in MethylC-analyzer to examine the DNA methylation status of these two genomes. Raw reads of three ADPKD and three non-ADPKD RRBS were mapped to the human hg19 reference genome with BS-Seeker2 (Guo et al. 2013).

ADPKD and non-ADPKD methylation are distinct, and two groups can be clearly observed in the PCA plot (Additional file 1: Fig. S1A). The clustering heatmap further showed a clear difference in methylation between ADPKD and non-ADPKD samples (Additional file 1: Fig. S1B). The average CG methylation values in ADPKD and non-ADPKD tissues are shown in Additional file 1: Fig. S1C. While the percent methylation in ADPKD was 53.6% on average, that in non-ADPKD was 54.8%, indicating that ADPKD was hypomethylated by 1.2% in comparison to the non-ADPKD reference genome. Additional file 1: Fig. S1D shows that the methylation levels of the three ADPKD samples were all lower than those of the non-ADPKD samples on each chromosome. Additional file 1: Fig. S1E revealed that although the global differences in methylation levels (∆ methylation) between ADPKD and non-ADPKD were minor (1.2%, Additional file 1: Fig. S1C), highly variable CG methylation was observed locally in several chromosomal regions, indicating that ADPKD-derived genomic DNA presents global hypomethylation compared with non-ADPKD kidneys (Bowden et al. 2018). Overall, we found that ADPKD exhibited a lower average CG methylation level than non-ADPKD and that this trend persisted across the genome.

We then investigated the distribution of differential DNA methylation levels in a gene-centric manner for the two groups. Additional file 1: Fig. S1F shows that decreased CG methylation levels were observed at TSSs and TESs, whereas methylation was increased within the gene bodies, for both ADPKD and non-ADPKD. The differential methylation patterns observed in these two groups are shown in Additional file 1: Fig. S1G. The ADPKD samples exhibited generally decreased CG methylation in both gene bodies and surrounding regions relative to the non-ADPKD samples. We further identified the DMRs with respect to CG sites. A 20% difference in DNA methylation (p value < 0.01) within 500 bp was considered a significant DMR. In total, we identified 1751 DMRs in ADPKD compared to non-ADPKD, comprising 965 hyper- and 786 hypo-DMRs (Additional file 1: Fig. S1H). Then, we investigated whether these DMRs were localized to specific genomic regions or were randomly distributed. As shown in Additional file 1: Fig. S1I, CG DMRs exhibited strong enrichment in the 3’UTR after adjusting for RRBS fragments. In total, 939 genes were associated with DMRs at a promoter or gene body (Additional file 1: Fig. S1H) and were considered differentially methylated genes (DMGs). Among these DMGs, 933 were located in gene bodies and 134 were in promoters. Some of these DMGs were reported in an earlier study associated with ADPKD (Woo et al. 2014), for example, *PKD1* (a major driving gene in ADPKD), *NOTCH1* (which regulates the cell differentiation pathway), and *SLC22A18* (participating in cellular transport) were all hypermethylated in the gene body.

#### Feature comparison with other methylome analyzers

MethylC-analyzer is specifically designed for comparative DNA methylation analysis of post-alignment BS-seq data, including WGBS and RRBS. MethylC-analyzer provides comparisons of methylation at the scale of the whole genome (methylation levels along the whole genome by chromosome) and individual regions (identifying DMRs) and further interrogates the methylome around specific genomic features (i.e., gene bodies or promoters). Table 1 provides a comparison of the features offered by MethylC-analyzer and other published computational tools designed for the analysis of BS-seq, including BAT (Kretzmer et al. 2017), Bicycle (Grana et al. 2018), RnBeads 2.0 (Muller et al. 2019), HOME (Srivastava et al. 2019) and nf-core/methylseq (Ewels et al. 2020). Among the 5 tools that were evaluated, MethylC-analyzer includes most features except for raw read alignment. As there are already many useful bisulfite-specific aligners, such as BS Seeker2 (Guo et al. 2013), and their output files are mostly compatible with MethylC-analyzer, MethylC-analyzer actually allows users more flexibility to use their preferred aligner.

**Table 1:** Comparisons of MethylC-analyzer with other methylation analysis tools

Features	MethylC-analyzer	BAT (2017)	Bicycle (2018)	RnBeads 2.0 (2019)	HOME (2019)	nf-core/methylseq (2020)
Environment	CLI/GUI/ Docker	Docker	CLI	CLI/GUI	CLI	Docker
Experimental approaches	WGBS, RRBS	WGBS, RRBS	WGBS and 5hmC seq	EPIC microarrays, WGBS and RRBS	WGBS, RRBS	WGBS, RRBS
Alignment	−	+	+	−	−	+
Sequence context of cytosine methylation	CG, CHG, CHH	CG	CG, CHG, CHH	CG	CG, CHG, CHH	CG, CHG, CHH
Visualization of the general methylome	PCA, Hierarchical clustering heatmap	−	−	PCA, Hierarchical clustering heatmap	−	−
Genome-wide visualization	+	+	−	+	−	−
Visualization of methylation levels at specific regions	+	−	−	−	−	−
Differential methylation analysis	DMRs and DMGs	DMRs	DMRs	DMRs	DMRs	-
Visualization of DMR enrichment within specific genomic features	+	−	−	−	−	−
Converting GTF gene annotation to .bed files with 7 genomic regions	+	−	−	−	−	−
Generating files for loading into genome browsers (i.e., IgV)	+	−	+	−	−	−
DMR testing method	Student's t test	2D-KS with dynamic border	Likelihood ratio of beta-binomial models	Welch’s t test	Weighted logistic regression	−

"+", available; "−", not available

*CLI* command line interface, *GUI* graphical user interface, *WGBS* whole-genome bisulfite sequencing, *RRBS* reduced representation bisulfite sequencing, *5hmC* 5′ hydroxymethylcytosine, *PCA* principal component analysis, *DMRs* differentially methylated regions, *DMGs*, differentially methylated genes

In terms of the user interface, only bicycle and nf-core/methylseq lack a user-friendly interface to provide a simple deployment environment. For processing methylation data generated using different experimental approaches, all tools can process WGBS; MethylC-analyzer, BAT, RnBeads 2.0, and nf-core/methylseq can also be applied to RRBS; and microarray and hydroxymethylation data can be handled only by RnBeads 2.0 and Bicycle, respectively. Apart from nf-core/methylseq, most of these tools provide differential methylation analysis. In terms of visualization, MethylC-analyzer provides several unique functions to visualize the methylation analysis results, including whole-genome and region-specific plots, which are features that the other tools lack. In brief, MethylC-analyzer provides comprehensive functionalities to process WGBS and RRBS and allow versatile downstream analysis.

#### Comparison of DMR calling with other DMR software

**Fig. 3:**
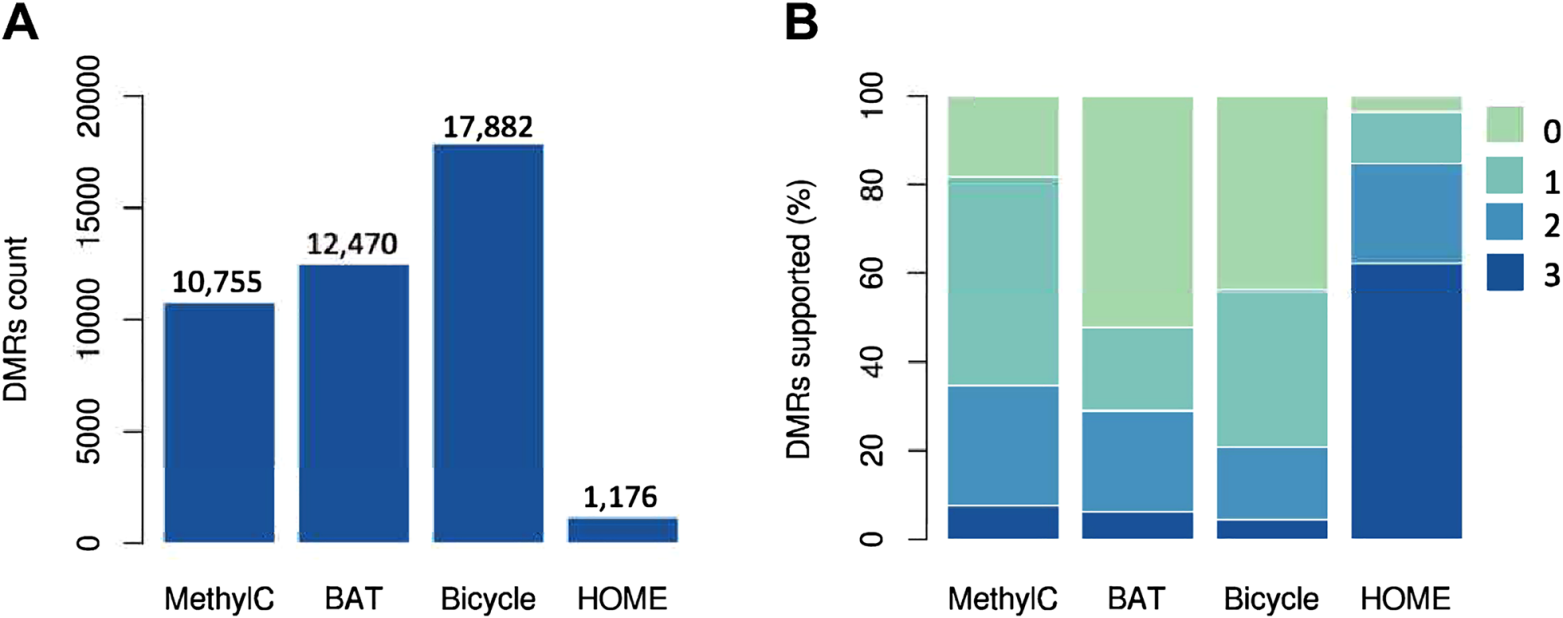
DMR calling comparison. **A** Number of CG DMRs called by MethylC-analyzer, BAT, Bicycle, and HOME using the Arabidopsis WGBS data. **B** The percentage of DMRs confirmed by the DMRs calling tools. The color key indicated the number of other DMR tools detected the same DMR. For example, "0" is the set of DMRs predicted by only one caller, and "3" is the set of DMRs predicted by all callers

We compared the DMRs identified by BAT, Bicycle, HOME, and MethylC-analyzer (Table 1). The test data were from Arabidopsis, comprising WGBS of 2 *otu5* mutant samples and 2 wild-type controls (Yen et al. 2017). Each tool was used with its default parameters to predict DMRs between *otu5* and WT strains. MethylC-analyzer, BAT and Bicycle were able to predict approximately 10–18 K DMR, whereas HOME was able to predict only approximately 1 K DMR (Fig. 3A), which may suggest a lower prediction sensitivity. For each tool, we also calculated the % DMRs that were confirmed by the other tools (Fig. 3B). It appears that 82% of the DMRs predicted by MethylC-analyzer were also confirmed by at least one other tool, higher than the proportions for BAT (48%) and Bicycle (56%). HOME reaches 96%, although the total number of predicted DMRs is very small.

In Additional file 2: Fig. S2, we present screenshots of the genome browser showing the predicted DMRs. The left and middle panels show the highly confident DMRs confirmed by multiple tools. The right panel shows a DMR that was predicted by MethylC-analyzer only and not by the other three tools. Overall, the results indicate that MethylC-analyzer might have a superior balance between sensitivity and specificity compared with the other tools.

## Conclusions

We presented MethylC-analyzer, which is specifically designed for analyzing post-alignment WGBS and RRBS data. MethylC-analyzer is capable of profiling BS-seq data to compare the DNA methylation between two datasets. Compared with other bioinformatics tools, MethylC-analyzer provides comprehensive analyses, including visualization of global methylation patterns, genome-wide and gene-centric methylation distribution, DMR identification, and DMR enrichment analysis, incorporating most of the features found in similar published tools. MethylC-analyzer also comes with a user-friendly GUI and useful tutorials that will enable biologists to evaluate DNA methylation more effectively.

## Supplementary Information

**Additional file 1: Fig. S1.** Visualization of the methylome analysis between Autosomal dominant polycystic kidney disease (ADPKD) and non-ADPKD. (A) PCA and (B) Hierarchical clustering showed clear difference between ADPKD and non-ADPKD. (C) The average methylation level in two groups in 3 contexts (CG, CHG, CHH) (D) The CG methylation level in ADPKD in genome-wide (E) Genome- wide plot of Δ methylation levels (ADPKD-nonADPKD). (F) Metagene plot of CG methylation levels. (G) Metagene plot of Δ CG methylation levels. (H) The summary of DMR and DMG numbers. (I)DMRs enriched in 3’UTRs and intergenic regions (IGR). TSS, transcription start site; TES, transcription end site; 5’UTR, 5’ untranslated region; CDS, coding sequence; 3’UTR, 3’ untranslated region.

**Additional file 2: Fig. S2.** Genome browser snapshots showing the CG methylation with differentially methylated regions between Wild-type and otu5 mutant (MT) plants using different DMR calling tools. (A) Common DMR regions with all 4 tools. (B) The DMR specifically called from MethylC-analyzer.

**Additional file 3: Table S1.** The example format of CG map.

## Data Availability

Publicly available datasets GSE110057, GSE122394, GSE148753 were analyzed in this study. Project name: MethylC-analyzer. Project home page: https://github.com/RitataLU/MethylC-analyzer. Operating system(s): Linux. Programming language: Python and R. Other requirements: Python = 3.9 and R > 3.6. License: GNU GPL. Any restrictions to use by non-academics: None.

## Abbreviations

WGBS: Whole-genome bisulfite sequencing
RRBS: Reduced representation bisulfite sequencing
EM-seq: Enzymatic methyl sequencing
MeDIP: Methylated DNA immunoprecipitation sequencing
TSS: Transcription start site
TES: Transcription end site
5’UTR: 5′Untranslated region
CDS: Coding sequence
3’UTR: 3′Untranslated region
GUI: Graphical user interface
ADPKD: Autosomal dominant polycystic kidney disease
DMR: Differentially methylated region
DMG: Differentially methylated gene
