# Correction: MethylC‑analyzer: a comprehensive downstream pipeline for the analysis of genome‑wide DNA methylation

**DOI:** 10.1186/s40529-023-00386-9

**Published:** 2023-05-30

**Authors:** Rita Jui‑Hsien Lu, Pei‑Yu Lin, Ming‑Ren Yen, Bing‑Heng Wu, Pao‑Yang Chen

**Affiliations:** 1 Institute of Plant and Microbial Biology, Academia Sinica, Taipei, 115 Taiwan; 2 Department of Medicine, Washington University in St. Louis, St. Louis, MO USA

**Correction: Botanical Studies (2023) 64:1** 10.1186/s40529-022-00366-5

In the original publication of the article (Lu et al. 2023) Fig. 3 was incorrectly published. The corrected Fig. 3 is given below.

**Fig. 3:**
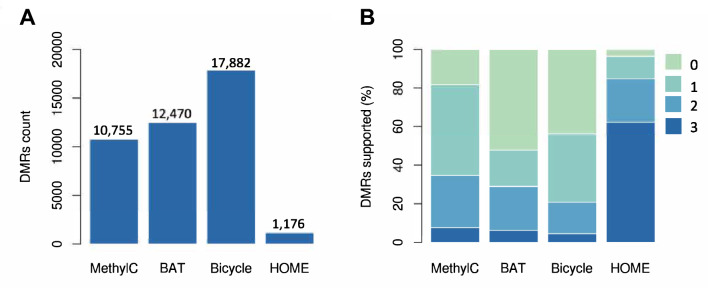
DMR calling comparison. **A** Number of CG DMRs called by MethylC-analyzer, BAT, Bicycle, and HOME using the Arabidopsis WGBS data. **B** The percentage of DMRs confirmed by the DMRs calling tools. The color key indicated the number of other DMR tools detected the same DMR. For example, "0" is the set of DMRs predicted by only one caller, and “3” is the set of DMRs predicted by all callers

The original article (Lu et al. 2023) has been corrected.
