# Supplementary material for: MethylC-analyzer: a comprehensive downstream pipeline for the analysis of genome-wide DNA methylation
Source: Bot Stud. 2023 Jan 6;64:1. doi: 10.1186/s40529-022-00366-5 (PMC9823188; doi:10.1186/s40529-022-00366-5)
Supplement: Supplementary file 1 — Additional file 1: Fig. S1. Visualization of the methylome analysis between Autosomal dominant polycystic kidney disease (ADPKD) and non-ADPKD. (A) PCA and (B) Hierarchical clustering showed clear difference between ADPKD and non-ADPKD. (C) The average methylation level in two groups in 3 contexts (CG, CHG, CHH) (D) The CG methylation level in ADPKD in genome-wide (E) Genome- wide plot of Δ methylation levels (ADPKD-nonADPKD). (F) Metagene plot of CG methylation levels. (G) Metagene plot of Δ CG methylation levels. (H) The summary of DMR and DMG numbers. (I)DMRs enriched in 3’UTRs and intergenic regions (IGR). TSS, transcription start site; TES, transcription end site; 5’UTR, 5’ untranslated region; CDS, coding sequence; 3’UTR, 3’ untranslated region. [file 40529_2022_366_MOESM1_ESM.pdf]

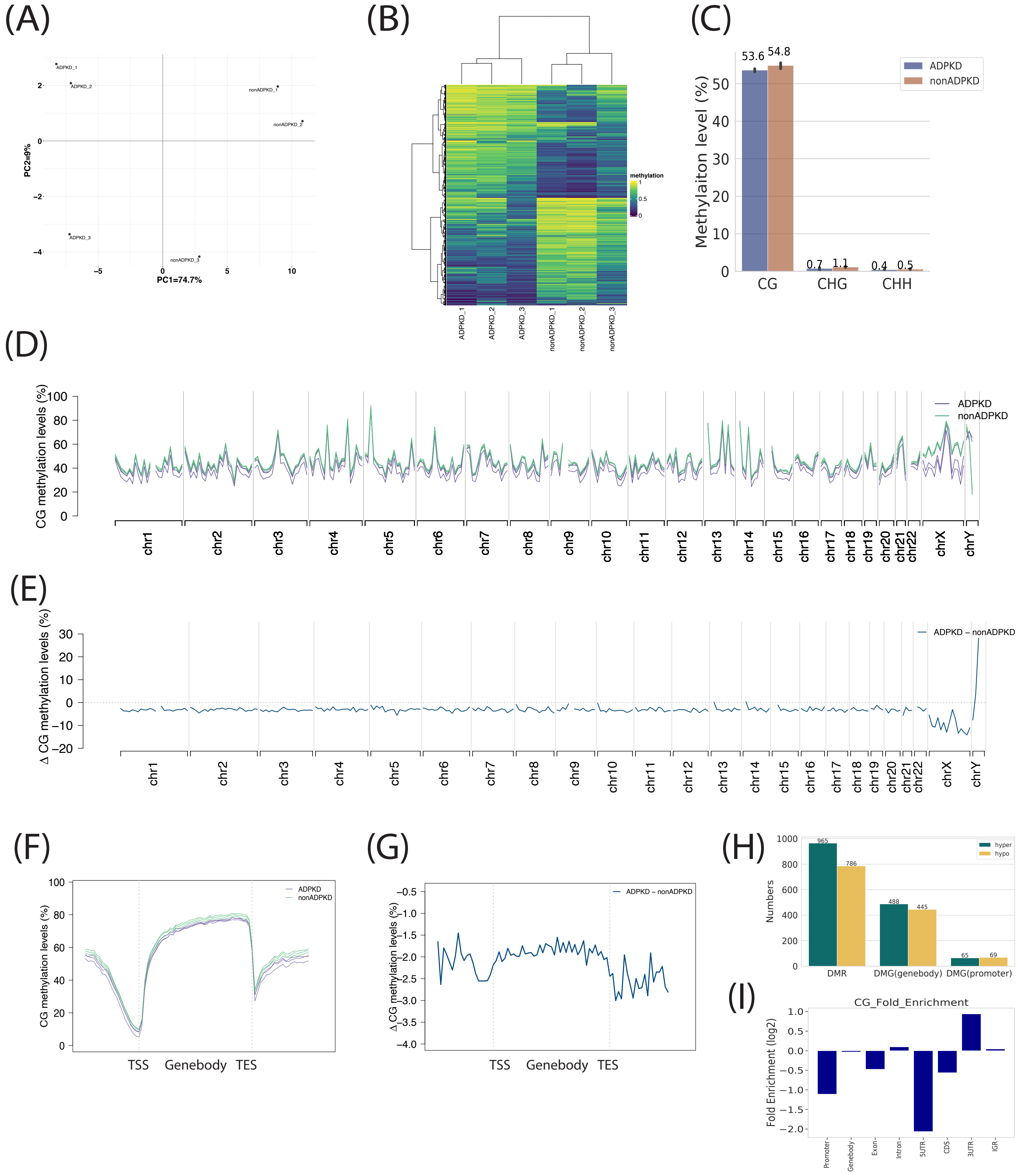

Figure S1. Visualization of the methylome analysis between Autosomal dominant polycystic kidney disease (ADPKD) and non-ADPKD.(A) PCA and (B) Hierarchical clustering showed clear difference between ADPKD and non-ADPKD. (C) The avarage methylaiton level in two groups in 3 context (CG, CHG, CHH) (D) The CG methylation level in ADPKD in genome-wide (E) Genome-wide plot of Δ methylation levels (ADPKD-nonADPKD). (F) Metagene plot of CG methylation levels. (G) Metagene plot of Δ CG methylation levels. (H) The summary of DMR and DMG nuumbers. (I)DMRs enriched in 3'UTRs and intergenic regions (IGR). TSS, transcription start site ; TES, transcription end site; 5'UTR, 5' untranslated region; CDS, coding sequence; 3'UTR, 3' untranslated region.
