# Supplementary material for: MethylC-analyzer: a comprehensive downstream pipeline for the analysis of genome-wide DNA methylation
Source: Bot Stud. 2023 Jan 6;64:1. doi: 10.1186/s40529-022-00366-5 (PMC9823188; doi:10.1186/s40529-022-00366-5)
Supplement: Supplementary file 2 — Additional file 2: Fig. S2. Genome browser snapshots showing the CG methylation with differentially methylated regions between Wild-type and otu5 mutant (MT) plants using different DMR calling tools. (A) Common DMR regions with all 4 tools. (B) The DMR specifically called from MethylC-analyzer. [file 40529_2022_366_MOESM2_ESM.pdf]

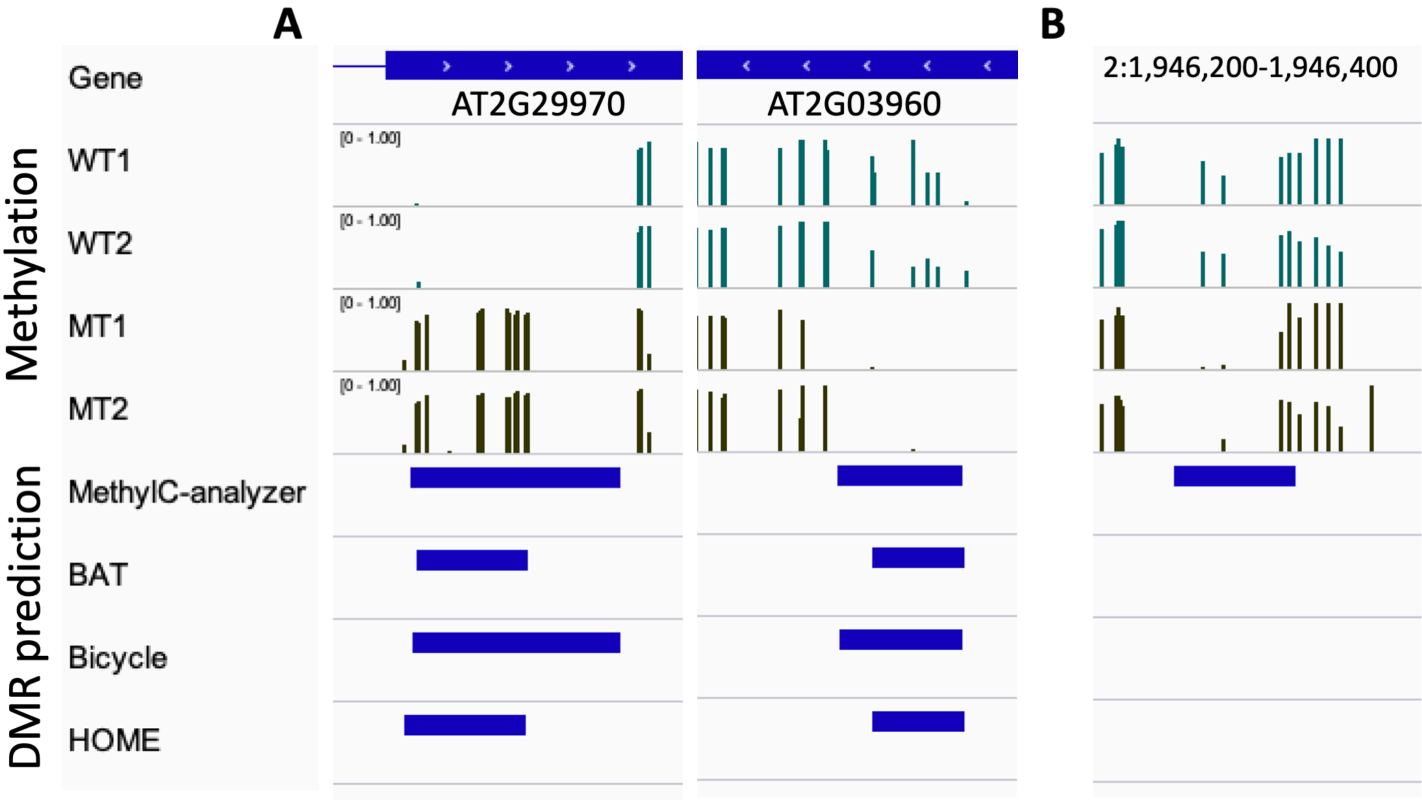

Figure S2. Genome browser snapshots showing the CG methylation with differentially methylated regions between Wild-type and *otu5* mutant (MT) plants using different DMR calling tools. (A) Common DMR regions with all 4 tools. (B) The DMR specifically called from MethylC- analyzer.
