# Supplementary material for: MethylC-analyzer: a comprehensive downstream pipeline for the analysis of genome-wide DNA methylation
Source: Bot Stud. 2023 Jan 6;64:1. doi: 10.1186/s40529-022-00366-5 (PMC9823188; doi:10.1186/s40529-022-00366-5)
Supplement: Supplementary file 3 — Additional file 3: Table S1. The example format of CG map. [file 40529_2022_366_MOESM3_ESM.pdf]

Table S1 The example format of CG map

```
chr1    G    3000851    CHH    CC  0.1    1    10
chr1    C    3001624    CHG    CA  0.0    0    9
chr1    C    3001631    CG     CG  1.0    5    5
chr1    G    3001632    CG     CG  0.9    9    10
```

Column Description

1	Chromosome name
2	The nucleotide on reference genome
3	1 based leftmost mapping position
4	context
5	Dinucleotide contex
6	Methylation level
7	Counts of reads support methylated Cytosine
8	Counts of reads support all Cytosine
